# Abundance and Expression of Shiga Toxin Genes in *Escherichia coli* at the Recto-Anal Junction Relates to Host Immune Genes

**DOI:** 10.3389/fcimb.2021.633573

**Published:** 2021-03-17

**Authors:** Zhe Pan, Yanhong Chen, Tim A. McAllister, Michael Gänzle, Graham Plastow, Le Luo Guan

**Affiliations:** ^1^ Department of Agricultural, Food and Nutritional Science, University of Alberta, Edmonton, AB, Canada; ^2^ Lethbridge Research Centre, Agriculture and Agri-Food Canada, Lethbridge, AB, Canada

**Keywords:** *Stx* gene, cattle breed, host immune genes, random forest model, Boruta algorithm

## Abstract

Shiga toxin (Stx) is the main virulence factor of Shiga toxin-producing *Escherichia coli* (STEC), and ruminants are the main reservoir of STEC. This study assessed the abundance and expression of *Stx* genes and the expression of host immune genes, aiming to determine factors affecting these measures and potential gene markers to differentiate *Stx* gene expression in the recto-anal junction of feedlot beef cattle. Rectal tissue and content samples were collected from 143 feedlot steers of three breeds (Angus, Charolais, and Kinsella Composite) over 2 consecutive years 2014 (n=71) and 2015 (n=72). The abundance and expression of *stx1* and *stx2* were quantified using qPCR and reverse-transcription-qPCR (RT-qPCR), respectively. Four immune genes (*MS4A1, CCL21, CD19*, and *LTB*), previously reported to be down-regulated in super-shedder cattle (i.e., > 10^4^ CFU g^-1^) were selected, and their expression was evaluated using RT-qPCR. The *stx1* gene abundance was only detected in tissue samples collected in year 2 and did not differ among breeds. The *stx2* gene was detected in STEC from all samples collected in both years and did not vary among breeds. The abundance of *stx1* and *stx2* differed (P < 0.001) in content samples collected across breeds (*stx1*:AN>CH>KC, *stx2*: AN=CH>KC) in year 1, but not in year 2. Expression of *stx2* was detected in 13 RAJ tissue samples (2014: n=6, 2015: n=7), while expression of *stx1* was not detected. Correlation analysis showed that the expression of *stx2* was negatively correlated with the expression of *MS4A1* (R=-0.56, P=0.05) and positively correlated with the expression of *LTB* (R=0.60, P=0.05). The random forest model and Boruta method revealed that expression of selected immune genes could be predictive indicators of *stx2* expression with prediction accuracy of *MS4A1* >*LTB* >*CCL21* >*CD19*. Our results indicate that the abundance of *Stx* could be affected by cattle breed and sampling year, suggesting that host genetics and environment may influence STEC colonization of the recto-anal junction of feedlot cattle. Additionally, the identified relationship between expressions of host immune genes and *stx2* suggests that the host animal may regulate *stx2* expression in colonizing STEC through immune functions.

## Introduction

Shiga toxin-producing *Escherichia coli* (STEC) cause foodborne disease that can lead to hemolytic uremic syndrome (HUS) and hemorrhagic colitis (HC) (Karmali et al., 1983). Approximately, 2.8 million acute illnesses around the world are attributed to STEC (Majowicz et al., 2014), with 60,000 of these occurring in the US annually (Scallan et al., 2011). Many infections in humans are attributed to direct or indirect contact with food or water contaminated with cattle feces (Mir et al., 2016). Ruminants, especially cattle are the main reservoir who are asymptomatic carriers of O157 and non-O157 STEC strains with the recto-anal junction (RAJ) as the main colonization site (Wang et al., 2016). Most *E. coli* strains are commensals within the gut of cattle (Mir et al., 2016; Wang et al., 2016), and are shed into the environment through feces. Cattle that shed more than 10^4^ CFU STEC per gram of feces are defined as “super-shedders” (SS), which are considered the primary source of STEC transmission on farms (Matthews et al., 2006). Although the incidence of *E. coli* O157:H7 causing disease in cattle is low, the prevalence of STEC including both *E. coli* O157:H7 and non-O157:H7 serotypes is not low in cattle ranging from 38.5%–75.0% (Cho et al., 2009). Both *E. coli* O157:H7 and non-O157:H7 serotypes can cause human disease and among non-O157 infections, up to 70% of human infections are attributed to six non-O157 STEC serogroups (O26, O45, O103, O111, O121, and O145) (Bosilevac and Koohmaraie, 2012).

Shiga toxins are the main virulence factors in STEC and other pathogenic bacterial species with the prototype toxins being designated as Shiga toxin 1a (Stx1a) and Shiga toxin 2a (Stx2a) (Melton-Celsa, 2014). These toxins differ in their virulence and host specificity (Fuller et al., 2011; Lee and Tesh, 2019; Petro et al., 2019) with Stx2 being most commonly associated with severe illness (HUS, hospitalization, and bloody diarrhea) in humans (Karmali et al., 1983; Panel et al., 2020). For example, 40% HUS, 41% hospitalization, and 43% bloody diarrhea cases reported in human were attributed to the detectable Stx2 (Panel et al., 2020). Therefore, identifying the abundance of *stx*1 and *stx*2 genes in cattle is important as they could harbor and shed STEC. However, information on the abundance and expression of *stx*1 and *stx*2 genes *in vivo* (e.g. in RAJ) of feedlot cattle is lacking. We hypothesize that the expression and abundance of *stx* genes at the RAJ is influenced by cattle breed and expression of host immune genes. Genetic variation in the host was found to be linked to the level of expression of immune genes in SS (Wang et al., 2018), which also affected the attachment and the colonization of the mucosa by STEC (Wang et al., 2018). The understanding of abundance and expression of *stx* genes in STEC from the main colonization site and its linkage with host immune gene expression will gain insights into the host-STEC interactions at the RAJ of feedlot cattle.

## Materials and Methods

### Animal Populations and Sample Collection

The animal trial followed Canadian Council of Animal Care Guidelines and was approved by the Animal Care and Use Committee, University of Alberta (Animal Care Committee protocol number AUP00000882). In total, rectal tissue and contents were collected over 2 consecutive years (2014 and 2015) from 143 cattle representing Angus breed (AN, n=47), Charolais breed (CH, n=48), and a crossbreed named Kinsella Composite (KC, n=48) that were reared at the University of Alberta Roy Berg Kinsella Research Station. Sampling was performed when animals were slaughtered at a comparable age (Year 2014: 492 days ± 30 days; Year 2015: 496 days ± 22 days; P=0.11) in each year. Ten cm^2^ rectal tissue was collected from RAJ and 10 ml rectum contents were squeezed from each steer within 30 min after slaughter at a federally approved abattoir. The samples were deep-frozen immediately in liquid nitrogen and stored at -80°C until use.

### DNA and RNA Extraction

Tissue and content samples of RAJ were ground into fine powder in liquid nitrogen and mixed homogeneously before DNA and RNA extraction. DNA was isolated from 0.1 g powdered tissue using repeated bead beating and a column (RBBC) method (Yu and Morrison, 2004) and purified using the QIAamp DNA Stool Mini Kit (Qiagen, Germany). The quantity and quality of DNA were assessed based on absorbance at 260 and 280 nm using the ND-1000 spectrophotometer (NanoDrop Technologies, Wilmington, USA). Trizol reagent (Invitrogen Corporation, Carlsbad, CA, USA) was used to isolate total RNA from 0.1 g powdered tissue following the manufacturer’s protocol. RNA was purified using the RNeasy MinElute Cleanup kit (Qiagen, Valencia, CA, USA). Quality and quantity of RNA were assessed using Agilent 2200 TapeStation (Agilent Technologies, Santa Clara, CA, USA) and Qubit 3.0 Fluorometer (Invitrogen, Carlsbad, CA, USA), respectively. DNA was extracted from 0.5 g of the RAJ contents from each steer using the same bead beating method described above. DNA was obtained from contents of 131 steers and were used for downstream analysis.

### Assessment of Shiga Toxin Gene Abundance Using qPCR

The DNA extracted from contents and tissues was used to evaluate the abundance of *Stx* genes using quantitative PCR (qPCR) with primers for the detection of all subtypes of *stx1* and *stx2* (**Table 1**) and SYBR Green I reagent (Fast SYBR green master mix; Applied Biosystems, Foster City, CA, USA). The qPCR was conducted in triplicates for each sample on a StepOnePlus™ Real-Time PCR System (Applied Biosystems, Foster City, CA, USA) with the program of one cycle at 95°C for 20 s followed by 40 cycles of 3 s at 95°C, 30 s at 60°C. Melting curve analysis with a temperature gradient of 0.1°C/s from 60 to 95°C with fluorescence signal measurement at 0.1°C intervals was performed to make sure targeted products were amplified specifically. The standard curve method was used to quantify *stx1* and *stx2* copy numbers. The standard curve was constructed by genomic DNAs isolated from strain *E. coli* FUA 1403 and *E. coli* FUA 1400, which contain *stx1* and *stx2*, respectively. The formula to calculate the absolute copy number of standard curves is described as follows (Li et al., 2009):

$$Absolute\;copy\;number\;\left(\frac{\#}{g\;Sample}\right)=\frac{Amount\left(\frac{g\;DNA}{g\;Sample}\right)*6.022*10^{23}\left(\frac{\#}{mol}\right)}{Length\;\left(bp\right)*660\left(\frac{g\;DNA}{mol*bp}\right)}$$

**Table 1:** Primer sequences, amplicon sizes, and annealing temperature for qPCR assays.

Genes	Oligo sequence (5’ to 3’)	Amplicon size, bp	Reference	Annealing temperature (°C)
*stx1*	F: GTCACAGTAACAAACCGTAACA R: TCGTTGACTACTTCTTATCTGGA	95	Jothikumar & Griffiths, 2002	60
*stx2*	F: ACTCTGACACCATCCTCT R: CACTGTCTGAAACTGCTC	118	He et al, 2020	60
*eae*	F: TGCTGGCATTTGGTCAGGTC R: CGCTGA(AG)CCCGCACCTAAATTTGC	175	Delmas et al, 2009	60
*CCL21*	F: GCTATCCTGTTCTCGCCTCG R: ACTGGGCTATGGCCCTTTTG	222	Wang et al, 2016	60
*LTB*	F: TGGGAAGAGGAGGTCAGTCC R: TAGCTTGCCATAAGTCGGGC	215	Wang et al, 2016	62
*CD19*	F: CTCCCATACCTCCCTGGTCA R: GCCCATGACCCACATCTCTC	127	Wang et al, 2016	64
*MS4A1*	F: GCGGAGAAGAACTCCACACA R: GGGTTAGCTCGCTCACAGTT	206	Wang et al, 2016	64
*β-actin*	F: CTAGGCACCAGGGCGTAATG R: CCACACGGAGCTCGTTGTAG	177	Malmuthuge et al, 2012	60

where 6.022 * 10^23^ represents the Avogadro’s constant (#/mol); Length (bp) is the length of template DNA; 660 represents the average mass of 1 bp double-strand DNA. The copy number of *stx1* or *stx2* was determined by relating threshold cycle (C_T_) values to standard curves based on the following regression formula (Li et al., 2009): Y = -3.193 * log X + 35.003 (Y, C_T_ value; X, copy number of 16S rRNA gene) (r^2^ = 0.996). The qPCR amplification efficiency was 88%–98%.

### Detection of Expression of *Stx* and Host Immune Genes Using qRT-PCR

Total RNA (0.1 µg) was further subjected to reverse transcription to synthesize cDNA using a cDNA Synthesis Kit (Bio-Rad, Hercules, CA, USA). Single-stranded cDNA was amplified using Oligo(dT)_12-18_ (Life Technologies, Carlsbad, CA, USA) and SuperScript™ II RT (Life Technologies, Carlsbad, CA, USA) was used to synthesize double-strand cDNA. Primers for the detection of *eae* expression are shown in **Table 1**. Quantitative RT-PCR of *stx1*, *stx2*, and *eae* was then performed using the double-strand cDNA and primers (**Table 2**) with the same thermal cycling program described above in triplicates for each sample. The expression of *stx1*, *stx2*, and *eae* was quantified by standard curve method described above.

**Table 2:** The prevalence analysis of *stx1* and *stx2* for samples collected from the rectal tissue and content in 2014 and 2015.

Sample type	Breed	Year 1 (2014)				Year 2 (2015)			
		No. (% ) Stx1-positive	P value	No. (%) Stx2-positive	P value	No. (%) Stx1-positive	P value	No. (%) Stx2-positive	P value
Tissue	AN	0 (0)*^a^*	1	23 (100)	1	24 (100)	1	24 (100)	1
	CH	0 (0)		24 (100)		23 (100)		23 (100)	
	KC	0 (0)		24 (100)		24 (100)		24 (100)	
	AN	18 (78)	0.001***	22 (96)	<0.001***	1 (6)	0.069	17 (94)	0.272
Content									
	CH	7 (35)		20 (100)		0 (0)		24 (100)	
	KC	6 (27)		4 (18)		4 (17)		24 (100)	

a Values presented here were numbers and percentages of Stx-positive samples. Fisher’s exact test was used to examine the differential prevalence of stx1 and stx2 among three breeds within each sample type. For comparisons, P-values were included along with the level of statistical significance (P ≤ 0.001***).

In addition, four genes reported to be differentially expressed between SS and non-shedding (NS) cattle (Wang et al., 2016); chemokine (C-C motif) ligand 21 (*CCL21*), lymphotoxin beta (*LTB*), CD19 molecule (*CD19*), and 4-domains, subfamily A, member 1 (*MS4A1*) were selected to study their relationship with Stx gene abundance and expression. The same qPCR amplification conditions were used for the four genes with their respective primers (**Table 1**). Four commonly used housekeeping genes, including bovine *GAPDH*, 18S rRNA genes*, RPLP0*, and the β-actin gene, were also quantified by qPCR (Wang et al., 2016). As β-actin exhibited the most consistent Cq value it was used as the house-keeping gene for evaluating relative gene expression. The relative expression of each gene (*stx1*, *stx2*, and immune genes) was measured by ΔCq value, which was calculated as (Wang et al., 2016):

$$\Delta C_{q}=C_{q}target\;genes-C_{q}reference\;gene$$

with a higher ΔCq representing the lower expression while a lower ΔCq indicating higher expression. The qPCR amplification efficiency was 88%–98%.

### Statistical Analysis

The PROC MIXED model in SAS (ver. 9.13; SAS Institute Inc., Cary, NC, United States) was used to analyze the *stx1* and *stx2* abundance as well as host gene expressions together with all potential 2- and 3-way interactions among breeds, years, and sample types. In this statistical model, breed, sample type, and year were analyzed as fixed effects with steers as the random effect. Interactions were removed from the model if they were not significant (P > 0.05). Least square means were compared using the Bonferroni mean separation method after the removal of insignificant interactions and the significance was considered at P <0.05. The difference of prevalence of *stx1* and *stx2* was analyzed using Fisher’s exact tests. Non-parametric Mann-Whitney U test in R (Mangiafico, 2020) was used to assess differences in host gene expression between Stx2+ (expressed) and Stx2- (not expressed) groups, with differences considered significant at P<0.05. Correlation analysis was performed based on Spearman’s rank correlation coefficient (R) to identify relationships between expression of *stx2* and host genes using the “ggcorrplot” package in R with significance at P<0.05.

Isomap, a novel method for nonlinear dimensional reduction (Tenenbaum et al., 2000), was applied to determine the effect of breed, and sampling year on the expression of immune genes and *stx2* using the “RDRToolbox” package in R. In addition, Davis-Bouldin-Index (DBIndex) was used to compute Euclidean metrics to validate the clustering patterns of the expression of immune genes and *stx2*, with the value ≤ 1 indicating a well-separated cluster (Davies and Bouldin, 1979). Correspondence analysis (CA) was used to identify relationships among expression patterns using the “FactoMineR” package (Ringrose, 1992; Tekaia, 2016).

### Identification of Potential Gene Markers for *Stx* Gene Expression Using Mathematic Models

The random forest model was used to identify predictive indicators for *stx2* expression with the “RandomForest” package in R. The host gene expression data were divided into two groups: *stx2+* (expressed) and *stx2-* (not expressed). Two-thirds of each group was used as training data, and the rest (one-third) was used for validation. The accuracy rate (number of samples recognized correctly/total number of samples) was calculated to determine the model classification performance. The mean decrease in accuracy was used to assess the importance of host genes as predictive indicators of *stx2* expression. Variables with high mean decrease in accuracy indicate the higher contribution as compared to variables with low mean decrease accuracy (Han et al., 2016). The area under the ROC curve (AUC) was calculated to assess the robustness of the prediction model with the criteria being excellent (0.9–1.0), good (0.8–0.9), fair (0.7–0.8), weak (0.6–0.7), or fail (0.5–0.6) (Zhang et al., 2016). Moreover, the Boruta method, a random forest-based feature selection with the ability to remove less informative features, was used as a supportive approach to perform this prediction using the “Boruta” package in R (Kursa and Rudnicki, 2010).

## Results

### Factors Affecting the Abundance and Prevalence of *stx1* and *stx2*

Sampling year significantly impacted the abundance and prevalence of *stx* genes identified in RAJ samples (P<0.01), therefore, the effect of breed on the prevalence and abundance of *stx1* and *stx*2 was analyzed separately for each year. The prevalence of *stx1* and *stx2* in tissue samples was not affected by breed in either year (**Table 2**). In year 1, the prevalence of *stx1* in contents was higher (P = 0.001, **Table 2**) in AN (n=18; 78%) compared to CH (n=7; 35%) and KC (n=6; 27%), and the prevalence of *stx2* was higher (P < 0.001, **Table 2**) in AN (n=22; 96%) and CH (n=20; 100%) than in KC (n=4; 18%). However, the prevalence of *stx1* and *stx2* in content samples collected in year 2 was not affected by breed (P*_stx1_* = 0.069, P*_stx2_* = 0.272, **Table 2**) with a tendency for breed to affect the prevalence of *stx1.*

The abundance of *stx1* and *stx2* was affected (P < 0.001) by sample type (tissue *vs*. contents) for both years (**Table 3**). An interaction effect between breed and sample type for the abundance of *stx1* and *stx2* was detected in year 1 (P*_stx1_*<0.001, P*_stx2_*<0.001, **Table 3**), but not in year 2 (P*_stx1_* = 0.28, P*_stx2_* = 0.12, **Table 3**). In year 1, the abundance of *stx1* in contents was affected by breed with its abundance higher in AN> CH> KC (P<0.001, **Table 3**), while its abundance in rectal tissue was under the detection limit (**Table 3**). For *stx2*, it was detected in both tissue and content samples in year 1 with no difference in the abundance of *stx2* in tissue samples (**Table 3**), but with the higher abundance in rectal contents of AN and CH as compared to KC steers (P<0.0001, **Table 3**). For year 2, the abundance of *stx1* or *stx2* did not differ among breeds for either tissue or contents (**Table 3**), with the abundance of *stx1* and *stx2* in tissue being higher compared to that in contents (P*_stx1_*<0.001, P*_stx2_*<0.001, **Table 3**), respectively.

**Table 3:** Abundance of *stx1* and *stx2* using q-PCR for samples collected from the rectal tissue and content in 2014 and 2015.

Year	Breed	AN		CH		KC		P-Value		
	Type	T	C	T	C	T	C	Breed	Type	Breed*Type
2014	*stx1*	N/D *^a^*	4.09 (5.20)	N/D	1.73 (5.79)	N/D	1.40 (5.47)	<0.0001***	<0.0001***	<0.0001***
	*stx2*	6.02 (0.08)	4.92 (1.01)	5.31 (0.05)	5.91 (0.22)	5.70 (0.05)	1.00 (4.65)	<0.0001***	<0.0001***	<0.0001***
2015	*stx1*	6.78 (0.02)	0.25 (1.11)	6.82 (0.03)	N/D	6.76 (0.03)	N/D	0.31	<0.0001***	0.28
	*stx2*	5.70 (0.02)	4.58 (1.58)	5.73 (0.03)	4.91 (0.20)	5.67 (0.03)	5.06 (0.31)	0.17	<0.0001***	0.12

a The value was presented as Mean (SE) after log_10_ transformation (gene copy numbers/g sample). T represents tissue samples, C represents contents. For content and tissue samples, the lowest abundance that can be detected corresponds to 200 (2.3 after log_10_ transformation) gene copies/g and 40 (1.5 after log_10_ transformation) gene copies/g, respectively. Therefore, stx gene abundance that lower than 2.3 log_10_(gene copies/g) and 1.5 log_10_(gene copies/g) for content and tissue samples was defined as “underdetermined” (“N/D”) which is assumed to be “0” in our analysis, respectively. For comparisons among different factors and among interaction effects, P-values were included along with the level of statistical significance (P ≤ 0.001***).

### Expression of *stx1* and *stx2* Associated With the Rectal Tissue of Beef Steers

Expression of bacterial *stx1* was not detected, and bacterial *stx2* (defined as *stx2+*) was only detected in mucosal tissue from 13 cattle (2014: n=6, 2015: n=7, **Table S1**). The expression of *stx2* was more common in KC (n=9; 70%) than in AN (n=2; 15%) and CH (n=2; 15%). The non-parametric Kruskal-Wallis test showed that *stx2* expression did not differ among breeds (ΔCq _AN_=5.04; ΔCq _CH_=5.11; ΔCq _KC_=5.04; P_=_ 0.31), but there was a trend for difference between sampling years (ΔCq _Year 2014_ = 4.94; ΔCq _Year 2015_ = 5.15; P = 0.06).

### Expression of Selected Immune Genes in RAJ Tissue From Beef Steers

**Figure 1:**
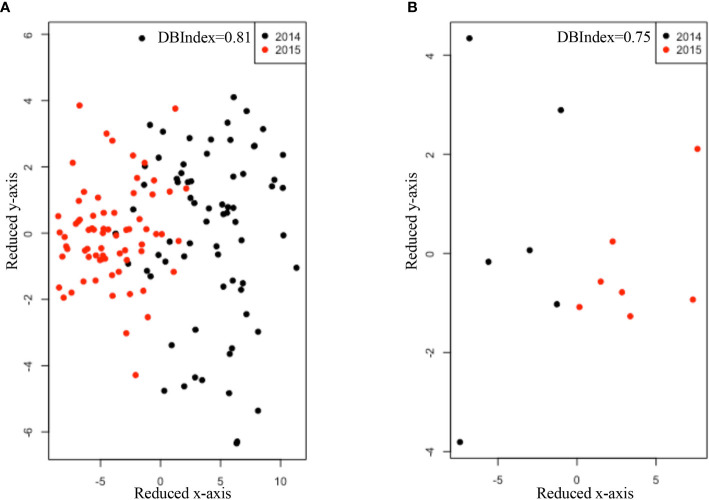
Comparisons of host gene expression patterns using non-parametric method Isomap and DBIndex value for sampling year effect **(A)** among all samples **(B)** as well as among Stx2+ samples. Black dots and red dots refer to samples collected in 2014 and 2015, respectively. DBIndex value was shown on the right corner of each figure. The lower DBIndex value, the well-separated cluster pattern.

In year 1, the expression of four selected immune genes was not affected by breed. In year 2, only expression of *CD19* and *CCL21* differed among breeds (P*_CD19_* = 0.02, P*_CCL21_* = 0.0035, **Table 4**). There was no difference (P*_MS4A1_* = 0.36, P*_CD19_* = 0.62, P*_CCL21_* = 0.94, P*_LTB_*=0.54, **Table 5**) in the expression of the four genes between *stx2+* and *stx2-* steers. Visually, host gene expression patterns from tissue samples were affected by year among all samples (Value_Year_ = 0.81, **Figure 1A**) as well as among *stx2+* samples (Value_Year_ =0.75, **Figure 1B**). However, host gene expression patterns did not differ among breeds based on DBIndex clustering value among all samples (Value_breed_=9.30, **Figure S1A**) or among *stx2+* samples (Value_breed_=1.64, **Figure S1B**).

**Table 4:** Quantification for relative expressions of four host gene among breeds and differed RFI using qRT-PCR for rectal tissue samples collected in 2014 and 2015.

Year	Immune genes	AN	CH	KC	P-Value
2014	*MS4A1*	2.80 (0.36)*^a^*	3.42 (0.29)	3.76 (0.44)	0.13
	*CD19*	-0.14 (0.38)	-0.32 (0.52)	-0.06 (0.35)	0.91
	*CCL21*	3.88 (0.45)	4.64 (0.35)	4.82 (0.46)	0.26
	*LTB*	-0.96 (0.48)	-0.97 (0.60)	-1.30 (0.44)	0.86
2015	*MS4A1*	3.76 (0.27)	3.65 (0.25)	4.26 (0.30)	0.26
	*CD19*	3.61 (0.28)	3.50 (0.29)	4.51 (0.24)	0.02*
	*CCL21*	5.94 (0.25)	4.90 (0.21)	5.87 (0.23)	0.0035***
	*LTB*	4.31 (0.44)	4.36 (0.40)	5.47 (0.36)	0.07

a The value was presented as Mean (SE) of ΔCq value that was calculated from each tissue sample under different year, breed, and feed efficiency. For comparisons among different factors and interaction effects, P-values were included with the level of statistical significance (P < 0.05*, P ≤ 0.001***).

**Table 5:** Expression differences for four host genes between Stx2+ and Stx2- samples using non-parametric Mann-Whitney U test.

Immune genes	Mean		Z-score	P-Value
	Stx2-	Stx2+		
*MS4A1*	3.65	3.44	0.92	0.36
*CD19*	1.90	1.54	0.49	0.62
*CCL21*	5.02	5.04	0.08	0.94
*LTB*	1.90	1.30	0.61	0.54

For comparisons between Stx2+ and Stx2- group, P > 0.05 indicates no significant difference.

### Association Between Expressions of *stx2* and Host Immune Genes

**Figure 2:**
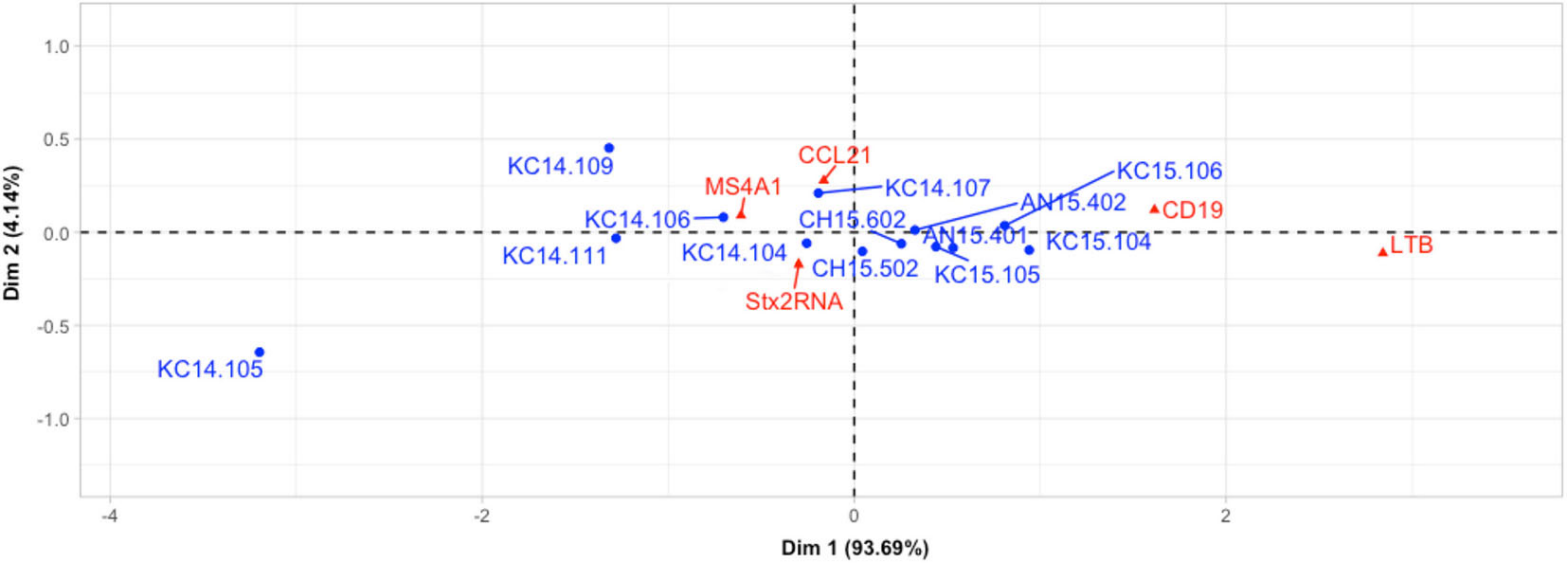
Assessment of associations between host immune gene expressions and Stx2+ samples using correspondence analysis. Red triangles and blue dots refer to host genes and Stx2+ samples, respectively. For example, “AN14.105” means the number of this sample is 105, breed is Angus, and was collected in 2014.

Expression of *stx2* was negatively correlated with the expression of *MS4A1* (R=-0.56, P=0.05, **Table 6**) and positively correlated with the expression of *LTB *(R=0.60, P=0.05, **Table 6**). Neither *CD19* nor *LTB* clustered with *Stx2+* samples but *CD19* and *LTB* were positively correlated (R=0.98, P=0.00, **Table 6**). Correspondence analysis revealed that most of the samples (12 out of 13, outlier: KC14.105) grouped together in the CA plot with *MS4A1* and *CCL21* (**Figure 2**). In the correspondence analysis (CA), Dimension 1 (Dim1) represented up to 94% of the importance with *CD19* and *LTB* contributing the most to Dim1, with Dim2 only representing 4.14% of the variation (**Figure 2**).

**Table 6:** Correlation analysis among relative expressions of host genes and *stx2* expression among Stx2+ samples.

		Stx2RNA	*MS4A1*	*CD19*	*CCL21*	*LTB*
Stx2RNA	R-Value	1.00	-0.56	0.51	-0.44	0.60
	P-Value	0.00	0.05*	0.08	0.13	0.03*
*MS4A1*	R-Value		1.00	-0.55	0.39	-0.56
	P-Value		0.00	0.05*	0.19	0.05*
*CD19*	R-Value			1.00	0.19	0.98
	P-Value			0.00	0.53	0.00***
*CCL21*	R-Value				1.00	0.09
	P-Value				0.00	0.78
*LTB*	R-Value					1.00
	P-Value					0.00

R-value was defined as the correlation coefficient ranged from -1 to 1. For correlations with different genes, P-values were included along with the level of statistical significance (P ≤ 0.05*, P ≤ 0.001***).

### Prediction Model to Discover Potential Gene Markers for *stx2* mRNA Abundance

**Figure 3:**
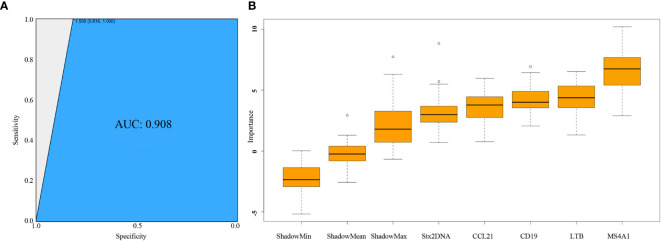
Assessment of Random Forest model using ROC curve and Boruta method. **(A)** Assessment of classification performance of random forest model using area under ROC (AUC). Sensitivity (y-axis) represents the fraction of samples with positive *Stx2* expression that the test correctly identifies as positive. Specificity (x-axis) represents the fraction of samples without *Stx2* expression that the test correctly identifies as negative. **(B)** Rank of host immune genes as markers for *Stx2* expression prediction using Boruta method. ○ represents the outliers in each Z-score.

Further analysis using a random forest model classifier based on expressions of four host immune genes *MS4A1, LTB, CCL21, CD19* revealed the accuracy for predicting *stx2* mRNA abundance was 96.5% for the training data and 93.6% for the validation data. The AUC value of 0.908 for the ROC curve also revealed a high accuracy and a robust prediction (**Figure 3A**). As an indicator of *stx2* expression, the prediction accuracy of *MS4A1, LTB, CCL21, CD19* was 47.55%, 45.35%, 41.44%, 36.80%, respectively. Further Boruta analysis also revealed that all four immune genes were attributes for *stx2* expression, with the ranking *MS4A1 > LTB = CD19 > CCL21* (**Figure 3B**).

## Discussion

This study characterized the abundance, prevalence, and expression of the *stx1* and *stx2* at the recto-anal junction in feedlot steers of three breeds over 2 consecutive years. Several studies have quantified the copy number of *stx1* and *stx2* in cattle feces using qPCR, with estimates ranged from 0 to 5.6 log_10_(gene copies/g) (Imamovic and Muniesa, 2011; Verstraete et al., 2014). Our estimates of the copy number of *stx1* and *stx2* in contents are within these ranges, with 1.24 to 4.13 log_10_(gene copies/g) (year 1, *stx1*), 0 to 0.45 log_10_(gene copies/g) (year 2, *stx1*), 0.86 to 5.38 log_10_(gene copies/g) (year 1, *stx2*), and 4.51 to 5.09 log_10_(gene copies/g) (year 2, *stx2*). However, there was a markable difference in the copy number of *stx* in tissue samples when compared to RAJ contents. Stx genes associated with RAJ tissue samples ranged from 5.62 to 6.07 log_10_(gene copies/g) (year 1, *stx2*), 6.71 to 6.85 log_10_(gene copies/g) (year 2, *stx1*), and 5.61 to 5.76 log_10_(gene copies/g) (year 2, *stx2*). We speculate that the high *stx* copy numbers detected from tissues likely represents the higher possibility of the STEC colonization on RAJ mucosa. Indeed, a previous study has reported that the abundance of *E. coli* O157 strain was inconsistent between RAJ tissues and content samples (Keen et al., 2010), suggesting that Stx carrying bacteria were associated with the epithelium of RAJ in the steers in addition to their presence in digesta. Based on our results, digesta samples only present a proportion of the actual STEC that inhabit in the RAJ of cattle, with the higher population directly colonizing epithelial tissue. These suggest that it should include fecal samples together with rectal mucosa swabs or biopsies to have more accurate estimation of *stx* gene abundance in cattle.

Our study further revealed that the abundance and prevalence of the *stx* genes was affected by breed and sampling year, and such effects were *stx* type dependent. However, a previous study found no relationship between cattle breed and the presence of *stx* at the RAJ (Mir et al., 2016). The inconsistency between our and previous findings may be due to differences in breed, age [calf (Mir et al., 2016) *vs*. steer], and diets of the cattle. In this study, Angus, Charolais, and Kinsella Composite breeds were used to examine the abundance and prevalence of *stx* genes, while previous studies collected samples from hybrid Angus-Brahman beef calves (Mir et al., 2016). Steers in our study were fed a high gain diet and slaughtered at similar body weight, but still differed in *stx1* and *stx2* prevalence across breeds, suggesting the highly individualized response to STEC colonization. Therefore, host genetics may alter the gut environment through influences on immunity and the microbiome (Wang et al., 2018), which may influence the prevalence of STEC and the prevalence and abundance of *stx* genes in the samples. The observed differences between sampling years suggest that environmental factors together with host genetics impact the prevalence of the *stx* genes in the RAJ of steers. Higher ambient temperatures have been shown to be associated with increased prevalence of both *stx1* and *stx2* in the rectal mucosa of both dairy and beef cattle (Fernandez et al., 2009; Tahamtan et al., 2010). For our study, the average ambient temperatures were similar between the two years (3.25°C for 2014 *vs*. 5.63°C for 2015) and as a result it is unlikely to account for the difference in detection of *stx1* and *stx2* between years. Other ecological factors such as seasonality, water and soil sources, and factors associated with farm management may also contribute to varied STEC colonization. Future long term monitoring studies are needed to determine to what extent these environmental factors contribute to the prevalence of both *stx1* and *stx2* in the RAJ of cattle.

Although the presence of both *stx1* and *stx2* genes were detected, only expression of *stx2* was found in the RAJ tissue of beef steers. Severe STEC infections that result in HUS are mostly associated with *stx2* as its product is 400 times more toxic (as quantified by LD_50_ in mice) than the product of *stx1* (Riley et al., 1983). *Stx2*-producing *E. coli* strains were reported to be in 71% (34 out of 48) of children with HUS, while only 40% (four out of 10) of patients were associated with *stx1*-producing *E. coli* strains (Ludwig et al., 2001). It is noticeable that the prevalence of *stx2* gene expression in steers (8.5% for year 1, 9.7% for year 2) is similar to the reported super shedder rate [~10% (Matthews et al., 2006)], suggesting the expression of *stx2* might be highly correlated with super shedding (SS) and cattle with *stx2* expression might potentially be SS. Interestingly, all *stx2+* samples were from KC steers in 2014,suggesting KC might be more prominent carriers of STEC and further highlighting the role of breed.

We further speculate that the *stx2+* cattle may have higher colonization of STEC. As the adherence factor intimin encoded by *eae* gene enables STEC colonization (Farfan and Torres, 2012) and the presence of *eae* is correlated with the formation of attaching and effacing (A/E) lesions (Wieler et al., 1996) and *E. coli* O157:H7 colonization in bovine RAJ (Sheng et al., 2006), the expression of *eae* was also assessed in this study. The expression of *eae* was detected in nine out of 131 RAJ tissue samples (Data not shown). Of these, only two samples were *stx2* positive. A previous study isolated 326 *E. coli* strains from 304 fecal samples of clinically healthy wild boars, and found that 10 samples were *eae* positive belonging to different *E. coli* strains (Alonso et al., 2017). Besides, only one *stx2*+ *eae+ E. coli* strain (*E. coli* O145:H28) was characterized to date and was reported to be associated with HUS in human (Alonso et al., 2017). Although the occurrence of *eae*, alone or in combination with *stx2* is sporadic, diverse *E. coli* serotypes exist in beef cattle and among them certain serotypes could be potential human pathogens. Compared to previous studies only reported expressions of *eae* and *stx* from fecal samples, our study is the first to report expressions of these two genes on RAJ mucosa. The detection of *stx+*, *eae+*, and *stx2+eae+* cattle suggests the importance to include all serotypes instead of only *E. coli* O157:H7 for future SS research in practice to the prevention of SS transmission and the mitigation of potential human infections. Future study is needed to isolate *E. coli* serotypes who carry *stx+*, *eae+*, and *stx2+eae+* genes and evaluate their abundances in RAJ and feces of beef steers to verify whether they are SS. Although the abundance of O157 strains were not quantified in this study, our study highlights the importance to use marker genes to assess all STEC populations as opposed to only *E. coli* O157:H7. In addition to *eae* genes, Enterohemorrhagic *E. coli* autotransporters (Eha) A and B autotransporters that can colonize on bovine epithelia are vital adhesin factors in STEC and are higher prevalent among STEC strains (97% and 93%, respectively) (Wells et al., 2009; Easton et al., 2011). Particularly, *EhaA* promoted adhesion to primary epithelial cells of bovine RAJ and should be explored to identify relationships between *EhaA* and host immunity for fundamental understanding of host-STEC interactions and STEC colonization. Other adhesin factors that play a role in STEC colonization on bovine epithelia such as hemorrhagic coli pili (HCP), EspP rope-like fibers (Farfan and Torres, 2012) should also be explored to identify relationships between STEC adhesin factors and host immune gene expressions.

Previous studies have identified differences in the expression of *MS4A1, CD19, CCL21, LTB* genes at the RAJ of super-shedder *vs*. non-shedders (Corbishley et al., 2014; Wang et al., 2016). These genes are involved in B cell proliferation (Uchida et al., 2004), B cell receptor signaling pathway (Karnell et al., 2014), and the migration of B cells from bone narrow to lymphoid tissues (Bowman et al., 2000), as well as the induction of the inflammatory response system (Browning et al., 1995). The observed higher relative expression of *CD19* (a membrane co-receptor found on all B cells) in KC steers and the higher relative expression of *CCL21* in AN and KC than CH in 2015, suggests that expression of this gene in cattle is influenced by breed. Breed-driven gene expression against infections and biological processes have been explored in bovine tissues and cells. Examples include, the reduced expression of the *ALDOA* (Fructose-bisphosphate aldolase A) gene in the longissimus muscle of Wagyu- as compared to Piedmontese-sired offsprings (Lehnert et al., 2007), and the up-regulation of *CD9* (CD9 antigen) and *BoLA-DQB* (BoLa Class II histocompatibility antigen, DQB*101 beta chain) in the macrophage of Sahiwal compared to Holstein cattle in response to *Theileria annulate* infection (Glass and Jensen, 2007). In our previous study, the variation in expression of immune genes between SS and NS, could be due to the genetic variation (Wang et al., 2016), suggesting future genome wide association studies (GWAS) are needed to identify the genotypes and/or SNPs responsible for expression of immune genes that could directly or indirectly affect STEC colonization and expression of their virulence genes.

Lymphotoxin beta (LTB) induces the immune response and is crucial for the initiation of Lymphoid follicle (ILF) development (McDonald et al., 2005). Lymphoid follicles (ILFs) in the bovine rectum are regarded as the reservoir of secretory antibodies in the gut, serving as a frontline defensive system in the gastrointestinal (GI) tract (Tsuji et al., 2008). The positive correlation between *stx2* expression and relative expression of *LTB* suggests that cattle with higher *stx2* expression have lower *LTB* expression, which may lead to decreased production of lymphotoxin, reduced ILF development in the RAJ. Impaired ILF has been associated with a 10 to 100-fold increase in the colonization of *Enterobacteriaceae* in ileum of mice (Bouskra et al., 2008), and 100-fold increase in anaerobic bacteria in the small intestine of mice (Fagarasan et al., 2002). Also, a previous study indicated that super-shedders harbor a distinct fecal microbiota compared to non-shedder (Xu et al., 2014). These suggest that changes in *LTB* expression could lead to impaired ILF function and altered microbiota, which could promote STEC colonization in cattle. Expression of *MS4A1* was negatively correlated with *stx2* expression and *MS4A1* was in the dominant position of *stx2+* samples from the correspondence analysis, suggesting the vital role of *MS4A1* in regulating *stx2* expression and partially reflecting a strengthened adaptive immunity in *stx2+* cattle. *MS4A1* encodes CD20 which is expressed from late pro-B cells through memory cells with its function to enable optimal B cell immune response and against T-independent antigens (Kuijpers et al., 2010). Hence, these indicate that *MS4A1* is the key gene in connecting *stx2* expression to host adaptive immunity, and their negative correlation suggest the establishment of host recognition mechanisms for *stx2* expression.

To our knowledge, this study is the first to explore whether host gene markers were related to *stx* expression and potential STEC colonization using artificial intelligence-based approaches (Random Forest model and Boruta method). Based on results of mean decrease accuracy in the Random Forest Model and Boruta method and the biological functions of these four immune genes, our results highlight the relationship between host immune genes and *stx2* expression. Of the genes studied, *MS4A1* was the best predictor of *stx2* expression and it was in the *stx2+* sample cluster in the CA map. We used the non-parametric dimensionality reduction method, Isomap, to assess the relationship between the expression of host genes and *stx2*, and results supported the *stx2* expression is closely associated with host gene expression patterns. Isomap was initially developed for computational visual perception (Tenenbaum et al., 2000) and then used to investigate ecosystem crosstalk (Mahecha et al., 2007), human disease phenotypes, and gene expression (Dawson et al., 2005). Compared to principal component analysis (PCA), this approach is less restricted since it does not require any specific distribution (*i.e.* normal distribution) of data (Shlens, 2014). The clustering patterns generated by PCA were similar to Isomap results, which could be due to the limited number of genes analyzed. But the Isomap approach is suitable for mammalian studies since interactions among genetics, environment, and microbes are in nature nonlinear (Nicholson et al., 2004). Regardless, our previous studies have reported 57 differential expressed genes between SS and NS (Wang et al., 2018) and many genes are interplay in cattle to affect their immunity and microbiota, the complexity of gene-gene interactions should be taken into account for future studies. Further explorations to investigate more DE genes and their interactions either at the individual or whole transcriptome level could identify and verify the predictiveness of host genes as markers of *stx2* expression. In addition to the genetic background that alters the predictiveness of random forest model, mucosa attached microbes (bacteria and viruses) can also impact on host immune gene expressions which should also be considered for the future construction of the prediction model. Our previous study (Wang et al., 2018) identified relationships between RAJ mucosa-associated bacteria and expression of 19 out of 57 DE immune genes identified from SS compared to NS. Although four immune genes were not part of these 19 DE genes, future studies to include the expression of these genes are needed for the better understanding of STEC colonization and its relationship with host immune genes and model construction.

## Conclusion

Taken together, our results revealed that cattle genetic background (breed) and sampling year could affect the abundance and prevalence of STEC *stx1* and *stx2* genes in the RAJ of feedlot cattle. We identified the relationships between *stx2* expression and the expression of host immune genes, and found that *stx2* expression could be driven by expression of particular host immune genes (e.g., *MS4A1*). Our study established a model to correlate host gene expression to *stx2* expression, suggesting that its expression can be driven by the host. Although *Stx* detection from feces is a more direct method, the findings from this study revealed that it may not represent the true population of STEC colonized in RAJ which can be influenced by the tissue immune genes. Future studies are needed to elucidate mechanisms behind host-STEC interactions by applying methods including genome wide association studies (GWAS) that determines potential genetic variations related to host-STEC interactions and also explore digesta and mucosal attached microbiota variations to develop methods for the potential precise identification of STEC in cattle.

## Data Availability Statement

The raw data supporting the conclusions of this article will be made available by the authors, without undue reservation.

## Ethics Statement

The animal study was reviewed and approved by the Animal Care and Use Committee, University of Alberta.

## Author Contributions

ZP and YC performed experiments. ZP, MG, and LG were involved in experimental design and methodology development. ZP, TM, MG, and LG were involved in data analysis. ZP wrote the draft manuscript. TM, MG, GP, and LG contributed to manuscript revisions. GP, TM, and LG were involved in securing the funding for the project. All authors contributed to the article and approved the submitted version.

## Conflict of Interest

The authors declare that the research was conducted in the absence of any commercial or financial relationships that could be construed as a potential conflict of interest.
