# Supplementary material for: Abundance and Expression of Shiga Toxin Genes in Escherichia coli at the Recto-Anal Junction Relates to Host Immune Genes
Source: Front Cell Infect Microbiol. 2021 Mar 17;11:633573. doi: 10.3389/fcimb.2021.633573 (PMC8010187; doi:10.3389/fcimb.2021.633573)
Supplement: Supplementary file 1 [file DataSheet_1.pdf]

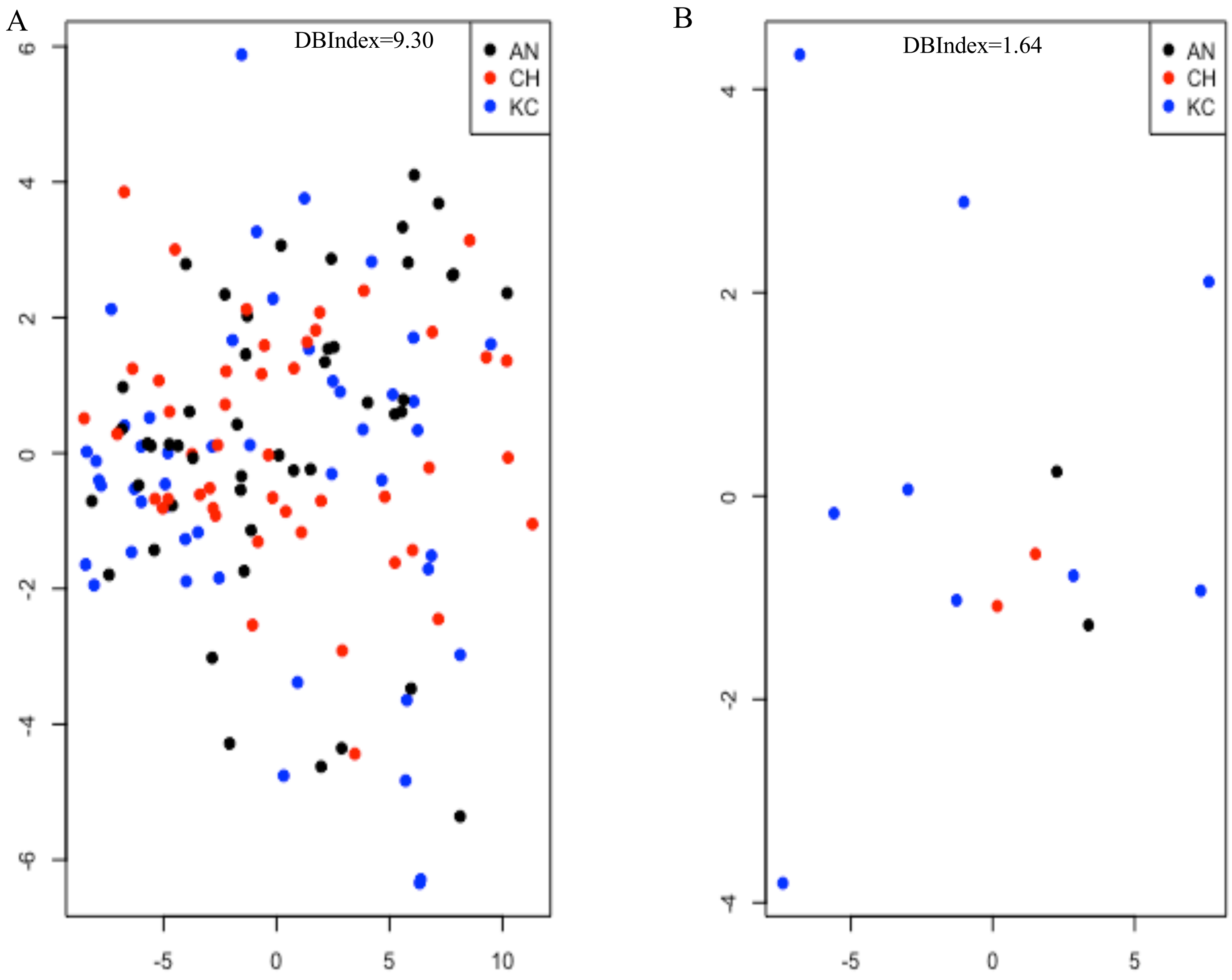

Figure S1. Comparisons of host gene expression patterns using non-parametric method Isomap and DBIndex value for breed effect among all samples (Fig S1A) as well as among Stx2+ samples (Fig S1B). Each dot represents a sample with black dots, red dots and blue dots representing Angus, Charolais, and Kinsella Composite breed. DBIndex value was shown on the right corner of each figure. The lower DBIndex value, the well-separated cluster pattern.

Table S1. Profiles of positive Stx2 expression samples including sample ID,year, and breed.

Sample ID	Breed	Stx2RNA (log_{10} transformation)
**2014-104**	KC	5.07
**2014-105**	KC	5.05
**2014-106**	KC	5.01
**2014-107**	KC	4.78
**2014-109**	KC	4.63
**2014-211**	KC	5.11
**2015-104**	KC	5.13
**2015-105**	KC	5.34
**2015-106**	KC	5.29
**2015-401**	AN	5.11
**2015-402**	AN	5.23
**2015-502**	CH	5.04
**2015-602**	CH	4.93
